# Supplementary material for: Multi‐dataset identification of innovative feature genes and molecular mechanisms in keratoconus
Source: J Cell Mol Med. 2024 Sep 19;28(18):e70079. doi: 10.1111/jcmm.70079 (PMC11412914; doi:10.1111/jcmm.70079)
Supplement: Supplementary file 2 — Material S2. [file JCMM-28-e70079-s002.zip › 7.GSEA analysis/5.RNF217-AS1/KEGG_INOSITOL_PHOSPHATE_METABOLISM.html]

Details for gene set KEGG\_INOSITOL\_PHOSPHATE\_METABOLISM[GSEA]

|  || Dataset | RNF217-AS1.group.cls#h\_versus\_l |
| Phenotype | group.cls#h\_versus\_l |
| Upregulated in class | l |
| GeneSet | KEGG\_INOSITOL\_PHOSPHATE\_METABOLISM |
| Enrichment Score (ES) | -0.40238047 |
| Normalized Enrichment Score (NES) | -1.537696 |
| Nominal p-value | 0.053608246 |
| FDR q-value | 0.35954115 |
| FWER p-Value | 0.712 |
Table: GSEA Results Summary

  

Fig 1: Enrichment plot: KEGG\_INOSITOL\_PHOSPHATE\_METABOLISM      
 Profile of the Running ES Score & Positions of GeneSet Members on the Rank Ordered List

  

| SYMBOL | TITLE | RANK IN GENE LIST | RANK METRIC SCORE | RUNNING ES | CORE ENRICHMENT || 1 | INPP4B | na | 2823 | 0.299 | -0.0382 | No |
| 2 | SYNJ2 | na | 6226 | 0.176 | -0.1109 | No |
| 3 | PIK3CD | na | 6778 | 0.159 | -0.1038 | No |
| 4 | ITPKA | na | 6807 | 0.158 | -0.0817 | No |
| 5 | PLCB3 | na | 7444 | 0.141 | -0.0797 | No |
| 6 | IPMK | na | 7992 | 0.128 | -0.0770 | No |
| 7 | PIKFYVE | na | 8727 | 0.111 | -0.0821 | No |
| 8 | PIP5K1A | na | 9172 | 0.102 | -0.0802 | No |
| 9 | PLCD1 | na | 9845 | 0.089 | -0.0868 | No |
| 10 | INPP1 | na | 11201 | 0.067 | -0.1161 | No |
| 11 | PIK3CB | na | 11439 | 0.064 | -0.1138 | No |
| 12 | PLCD3 | na | 12883 | 0.044 | -0.1491 | No |
| 13 | PLCG1 | na | 13642 | 0.035 | -0.1659 | No |
| 14 | PIK3CA | na | 14021 | 0.031 | -0.1724 | No |
| 15 | IPPK | na | 15968 | 0.009 | -0.2272 | No |
| 16 | MINPP1 | na | 16669 | 0.002 | -0.2471 | No |
| 17 | PLCZ1 | na | 16755 | 0.001 | -0.2494 | No |
| 18 | PI4KA | na | 16869 | -0.000 | -0.2527 | No |
| 19 | PIK3C2A | na | 16932 | -0.001 | -0.2543 | No |
| 20 | INPPL1 | na | 17188 | -0.003 | -0.2612 | No |
| 21 | INPP5K | na | 17197 | -0.004 | -0.2609 | No |
| 22 | SYNJ1 | na | 17629 | -0.007 | -0.2723 | No |
| 23 | IMPA1 | na | 18555 | -0.016 | -0.2966 | No |
| 24 | PIP5K1C | na | 18811 | -0.018 | -0.3013 | No |
| 25 | TPI1 | na | 19177 | -0.022 | -0.3087 | No |
| 26 | INPP5E | na | 19291 | -0.023 | -0.3086 | No |
| 27 | PTEN | na | 19758 | -0.028 | -0.3181 | No |
| 28 | INPP4A | na | 19981 | -0.030 | -0.3202 | No |
| 29 | PIP4K2B | na | 20260 | -0.033 | -0.3235 | No |
| 30 | ALDH6A1 | na | 20921 | -0.039 | -0.3369 | No |
| 31 | INPP5A | na | 21183 | -0.042 | -0.3384 | No |
| 32 | PLCB4 | na | 21374 | -0.044 | -0.3375 | No |
| 33 | PIP4K2C | na | 21562 | -0.046 | -0.3363 | No |
| 34 | PI4KB | na | 21741 | -0.048 | -0.3345 | No |
| 35 | PIK3C3 | na | 22999 | -0.062 | -0.3618 | No |
| 36 | PIK3C2B | na | 23777 | -0.072 | -0.3739 | No |
| 37 | OCRL | na | 24140 | -0.077 | -0.3732 | No |
| 38 | PLCG2 | na | 24806 | -0.087 | -0.3797 | No |
| 39 | INPP5B | na | 25592 | -0.100 | -0.3880 | Yes |
| 40 | PIP4K2A | na | 25647 | -0.101 | -0.3750 | Yes |
| 41 | ITPK1 | na | 26034 | -0.108 | -0.3706 | Yes |
| 42 | IMPA2 | na | 27058 | -0.127 | -0.3818 | Yes |
| 43 | CDIPT | na | 27382 | -0.134 | -0.3718 | Yes |
| 44 | PLCD4 | na | 28431 | -0.160 | -0.3789 | Yes |
| 45 | MIOX | na | 29152 | -0.180 | -0.3736 | Yes |
| 46 | ITPKB | na | 29842 | -0.201 | -0.3644 | Yes |
| 47 | INPP5J | na | 30054 | -0.208 | -0.3405 | Yes |
| 48 | PIK3CG | na | 30335 | -0.218 | -0.3172 | Yes |
| 49 | PIK3C2G | na | 31109 | -0.246 | -0.3039 | Yes |
| 50 | PLCB2 | na | 31149 | -0.248 | -0.2693 | Yes |
| 51 | ISYNA1 | na | 31847 | -0.282 | -0.2486 | Yes |
| 52 | PLCB1 | na | 32371 | -0.315 | -0.2183 | Yes |
| 53 | PLCE1 | na | 34473 | -0.637 | -0.1870 | Yes |
| 54 | PIP5K1B | na | 34727 | -1.347 | 0.0000 | Yes |
Table: GSEA details [plain text format]

  

Fig 2: KEGG\_INOSITOL\_PHOSPHATE\_METABOLISM      
 Blue-Pink O' Gram in the Space of the Analyzed GeneSet

  

Fig 3: KEGG\_INOSITOL\_PHOSPHATE\_METABOLISM: Random ES distribution      
 Gene set null distribution of ES for **KEGG\_INOSITOL\_PHOSPHATE\_METABOLISM**
